# Detection of Human papillomavirus and the role of p16^INK4a^ in colorectal carcinomas

**DOI:** 10.1371/journal.pone.0235065

**Published:** 2020-06-25

**Authors:** Larisse Silva Dalla Libera, Thalita de Siqueira, Igor Lopes Santos, Jéssica Enocencio Porto Ramos, Amanda Xavier Milhomen, Rita de Cassia Gonçalves de Alencar, Silvia Helena Rabelo Santos, Megmar Aparecida dos Santos Carneiro, Rosane Ribeiro Figueiredo Alves, Vera Aparecida Saddi

**Affiliations:** 1 Universidade Federal de Goiás (UFG), Programa de Pós-Graduação em Ciências da Saúde (PPGCS), Faculdade de Medicina (FM) e Instituto de Patologia Tropical e Saúde Pública (IPTSP), Goiânia, GO, Brasil; 2 Pontifícia Universidade Católica de Goiás (PUC/GO), Programa de Pós-Graduação em Ciências Ambientais e Saúde (MCAS) e Escola de Ciências Médicas, Farmacêuticas e Biomédicas (ECMFB), Goiânia, GO, Brasil; 3 Departamento de Patologia, Hospital Araújo Jorge (HAJ)—Associação de Combate ao Câncer em Goiás (ACCG), Goiânia, GO, Brasil; Istituto Nazionale Tumori IRCCS Fondazione Pascale, ITALY

## Abstract

**Introduction:**

Human papillomavirus (HPV) infection is associated with the development of anogenital and head and neck cancers. In recent years a potential role of HPV in colorectal cancer (CRC) has been suggested.

**Objective:**

To investigate the presence of HPV in colorectal carcinomas and to study the role of p16^INK4a^ as a marker of transcriptionally active HPV infection. In addition, to investigate the correlation between these findings and the CRC prognostic factors.

**Methods:**

Case control study with 92 cases of colorectal cancers, 75 controls of normal tissue adjacent to the tumor, and 30 controls of precursor lesions, including polyps and colorectal adenomas. Paraffinized samples were used, HPV detection and genotyping were performed by PCR and reverse hybridization by using the INNO LIPA kit, with SPF10 plus primers. The expression of the p16^INK4a^ protein was investigated using immunohistochemistry. Data analysis was performed using descriptive, univariate statistics and survival curves were calculated by using the Kaplan Meier and log-rank method.

**Results:**

HPV was detected in 13% of the cases and the most prevalent genotype was HPV 16. HPV DNA was not detected in either control groups. The high expression of p16^INK4a^ was observed in 30% of the cases, but it was not associated to the presence of HPV. The overall survival was 53.3% and was influenced by prognostic factors such as later stage, lymph node and distant metastasis.

**Conclusions:**

Based on these results, HPV is unlikely to be involved in colorectal carcinogenesis and p16^INK4a^ expression is not a relevant marker of transcriptionally active HPV infection in CRC.

## Introduction

Colorectal cancer (CRC) is the third most incident tumor and the fourth leading cause of cancer deaths worldwide [1]. According to the latest cancer mortality data in Brazil (2017), CRC is among the tumors that kill the most men (9,207 registered deaths) and women (9,660 registered deaths) [2]. In the United States, the relative five-year survival for CRC is 64.4% [3,4].

Genetic susceptibility plays a key role in a subset of CRC cases, but the vast majority of the tumors are sporadic and not inherited [5,6]. Several molecular pathways have been identified for CRC carcinogenesis [7–11], resulting in a classification of colorectal cancer into four molecular consensus subtypes (CMS1-4), each with a unique pattern of biology and gene expression [12–17].

Among the most studied genetic alterations in CRC are chromosomal instability; methylation profile; mutations in genes like APC (*adenomatous polyposis coli*), K-ras, p53, WNT, BRAF, MAD4, EGF; modifications in EGFR, P13-K, Map-kinase pathways and changes in mismatch repair [6,13,18–20].

Although epigenetic, environmental and immunological factors may additionally interact with these molecular pathways, there is still much uncertainties about the role of infectious agents in CRC [21].

Human papillomavirus (HPV) is the infectious agent most associated with mucoepithelial cancers. HPV infection is a necessary cause for cervical cancer and HPV is associated with other epithelial neoplasms, such as cancers of the oropharynx, penis, vulva, vagina and anus [22,23]. In these tumors the persistence of high-risk HPV infection is essential for the progression of premalignant lesions [24,25].

The integration of viral DNA into the cell genome leads to increased expression of the HPV, E6 and E7 oncogenes, as a result of the partial or total loss of the E2 gene, which is a transcriptional repressor for E6 and E7 [26–29]. Consequently, the overexpression of these oncoproteins leads to the degradation of p53 and pRb, inducing HPV carcinogenesis [26,30–33]. Inactivation of pRb by the virus proteins also leads to dysregulation of the cell cycle and increased expression of p16^INK4a^, an important tumor suppressor protein [34–37].

Even when there is no viral integration into the host cell genome, genetic or epigenetic changes are observed in the regulatory regions of the HPV E6 and E7 genes, demonstrating that the deregulation of these oncoproteins is crucial for viral infection-induced carcinogenesis [38].

The association of HPV with CRC has been investigated in several studies [39–43], however with controversial results. One of the main biases to prove such an association is the difficulty of studies demonstrating the presence of the virus in CRCs and precursor lesions, such as polyps and adenocarcinomas, and the absence of the virus in the adjacent normal tissue [41,43,44]. In addition, there are few studies that investigated the activity of E6 and E7 in CRC lesions, or even the integration of the viral genome in the host cell genome, crucial events to determine the causal role of HPV in CRC [39,40].

To understand the possible implications of HPV in colorectal carcinogenesis and to address some of the questions raised about the association of HPV with CRC, this study investigated the presence of HPV DNA in colorectal tumors, normal tissues, and precursor lesions for CRC. In addition, we further investigated the expression of the p16^INK4a^ protein in order to verify molecular changes potentially associated with the viral carcinogenesis.

## Materials and methods

### Ethical aspects and study design

This study was approved by the Research Ethics Committee of the Association for Combating Cancer in Goiás (*Associação de Combate ao Câncer em Goiás* CEP/ACCG) under protocol 1.856.467 (CAAE: 62214616.7.0000.0031).

**Fig 1:**
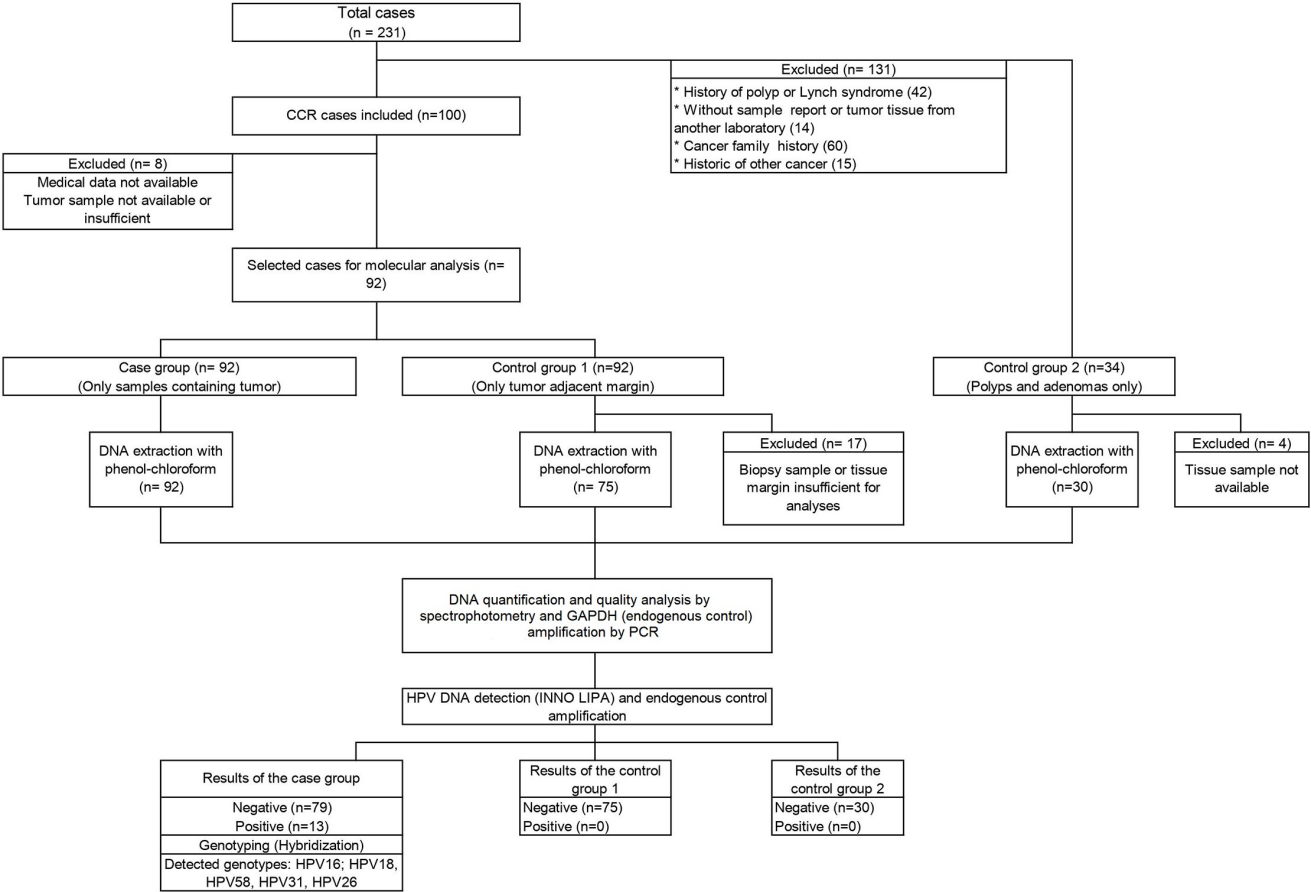
Flowchart of selection and analysis of the study groups.

This is a retrospective case-control study, including data collected from 92 patients with confirmed diagnosis of colorectal cancer from a referral hospital for cancer treatment in the Midwest of Brazil (Hospital Araújo Jorge in Goiânia, Goiás, Brazil), over a period of one year. Initially, a list of 231 patients diagnosed with colorectal cancer from 2008 to 2009 was consulted, all samples were anonymized, even before the researchers had access data. The patients' medical records were made available by the Medical Archive Sector and the samples and anatomopathological reports by the hospital's Pathology Laboratory. All information was collected between January 2017 and December 2017. After revision based on the inclusion criteria, 92 cases of colorectal cancers, 75 controls consisting of non-cancer tissue adjacent to the tumor, and 30 cases of precursor lesions, comprising polyps and colorectal adenomas, were included in the study. During the study there was no direct contact with the patients and their personal data weren’t necessary, therefore no informed consents from the patients were required. A description of the inclusion and exclusion criteria of the cases and the distribution of the study groups are presented in Fig 1.

The case group included participants with histopathological diagnosis of colon or rectal carcinoma, with clinical and pathological data available in the medical records and paraffin blocks available for analysis. Excluded cases were those whose primary tumor site in the colon or rectum was not confirmed, those who had a family history of cancer and or polyposis, cases that had received neoadjuvant chemotherapy or radiation therapy, or tumor biopsies whose tumor specimens were insufficient for the molecular analysis.

Two control groups were used for comparison. In the first control group, samples of non-cancer tissues adjacent to the patients' own tumors were included. This group was used to investigate whether the presence of HPV would be observed only in carcinomas or if the virus would also be present in tumor-free samples. The second control group consisted of samples of pre-neoplastic lesions, such as polyps and adenomas, without previous treatment. There was no matching criterion for this group, and its analysis was intended only to assess the role of HPV in the classic tumor progression model of colorectal cancer (polyp-adenoma-adenocarcinoma). Both the cases and the study controls were selected based on confirmation of the histopathological diagnosis. A survival analysis of patients diagnosed with colorectal cancer was also performed.

A sample size calculation was not performed since we aimed to include in the study all the cases that were diagnosed as colorectal cancer in the Pathology Department of the hospital. Our reference sample come from the registry of the Pathology Laboratory, so initially, a list of 231 patients diagnosed with colorectal cancer from 2008 to 2009 was consulted. After pathological/clinical review, 92 cases of colorectal cancers were considered eligible. A description of the inclusion and exclusion criteria of the cases is presented in Fig 1.

#### Data collection

All records were independently reviewed by two researchers and the information collected was registered in two databases, which were then compared and the inconsistencies resolved by a third researcher.

The sociodemographic characteristics of the patients included: gender; age at the time of diagnosis; ethnicity; marital status; habits such as smoking and drinking; family history of cancer, and history of neoplasia prior to colorectal carcinoma.

The clinical and pathological characteristics available included: tumor location; histological type; diagnostic method; treatment; tumor stage according to American Joint Committee on Cancer (AJCC) [45] and patient status at their last visit.

#### Analysis of samples

From the selected paraffin blocks, 5 μm sections were prepared by using a microtome. The sections were mounted on silanized slides for immunohistochemical analysis and placed in microtubes for DNA extraction and HPV detection. For each new block, the microtome was cleaned with 70% ethanol and the blades changed.

#### Molecular analysis

*DNA extraction*. All case samples were analyzed at the Genetic Diversity Laboratory of the Pontifical Catholic University of Goiás (PUC-GO). The tissue samples included in paraffin blocks were subjected to DNA extraction by the phenol/chloroform/isoamyl alcohol method, with a previous step of removing the paraffin with xylol and digestion with proteinase-K. The final DNA precipitation was done with isopropanol and DNA purification with 70% ethanol. As dewaxing of the sample can lead to tissue loss and consequent degradation of the DNA present in the sample [46], the amount of DNA extracted and its quality were evaluated by spectrophotometry (Thermo Scientific Nano Drop Products). The samples were then submitted to polymerase chain reaction (PCR), in order to amplify an endogenous control, a fragment of the human glyceraldehyde-3-phosphate dehydrogenase gene (GAPDH). Samples that were negative for the endogenous control were re-extracted. The DNA purification and detection protocols have been described in previous work by our group [47,48].

Samples outside the purity standards or without the amplification of the endogenous control gene were not considered for analysis and were subjected to DNA re-extraction when necessary. This procedure was introduced in order to avoid any bias in the DNA extraction methods, mainly in the dewaxing method, which may result in inhibition of proteinase K and decreased reliability of HPV detection [49–51]. The database with DNA concentration and purity ratios obtained for each sample is added as a supplementary material.

*Detection and genotyping HPV*. The detection of HPV DNA was performed by PCR with the commercial INNO-LiPA HPV Genotyping Extra II Amplification Kit (Fujirebio Europe^®^), which amplifies a conserved fragment of the L1 gene with 65 bp. The kit has the potential to amplify at least 54 HPV genotypes using SPF10 Plus primer oligonucleotides, which are highly sensitive and allow the simultaneous detection of several genotypes in a single sample. The kit also amplifies a positive control gene (endogenous human HLA-DBP1 gene).

Genotyping was performed by reverse hybridization using the INNO-LiPA HPV Genotyping Extra II Kit (Fujirebio Europe^®^). The assay allows the identification of 32 HPV genotypes, currently known as high risk genotypes [16, 18, 31, 33, 35, 39, 45, 51, 52, 56, 58, 59, 68], probable high risk [26, 53, 66, 70, 73, 82] and low risk [6, 11, 40, 42, 43, 44, 54, 61, 62, 67, 81, 83, 89].

The entire laboratory procedure, from sample handling, HPV detection and genotyping followed the international standards for HPV testing by the World Health Organization Laboratory Manual [52].

*Evaluation of the expression of p16*^*INK4a*^. The immunohistochemical analysis of p16^INK4a^ used the monoclonal antibody (CINtec® p16 Histology) and polymer-associated immunoperoxidase methods (Novolink-Novocastra Max Polymer Detection System—Leica).

The sections of colorectal tumors mounted on silanized slides were deparaffinized with xylol at room temperature and then rehydrated in a series of washes with alcohol (100%, 80% and 50% respectively) and phosphate buffer (PBS). The blocking of endogenous peroxidase was performed in 10V hydrogen peroxide at 3% for 10 minutes. Then the slides were washed under running water and immersed in PBS. Antigenic recovery was performed by moist heat and pressure in 10mM/pH 6.0 citrate buffer solution. After antigenic recovery, the slides were kept at room temperature for about 20 minutes and washed under running water. The blocking of endogenous peroxidase was performed in 10V hydrogen peroxide at 3% for 10 minutes, and then the slides were washed with running water and rinsed with phosphate buffer (PBS). After blocking, the slides were incubated at 4ºC, overnight, with the primary antibody, and then the slides were washed in running water and PBS and incubated for 30 minutes with the secondary antibody, being washed again in running water and PBS and incubated for 30 minutes with the dextran core polymer. After another wash with running water and PBS, the reaction was performed with 3–3'diaminobenzidine tetrahydrochloride, for 5 minutes, and the slides lightly counterstained with Harris hematoxylin. Then, the slides were dehydrated in alcohol and xylol and mounted with a coverslip using Entellan Novo (Merk). A positive control was included in each reaction performed, containing a case of invasive squamous cell carcinoma previously proved positive for p16^INK4a^. All reactions were processed under the same environmental conditions.

*Reporting and interpretation of p16*^*INK4a*^ *expression*. Evaluation of p16^INK4a^ expression was performed by a pathologist using an evaluation form for each sample. The analysis of p16^INK4a^ expression considered the labeling index of the respective protein in the nuclei and cytoplasms of the tumor cells, taking place in two stages. First, the area of the tumor with the highest number of marked cells (hot spots) was detected, then the number of positive cells was established semi-quantitatively in each hot-spot. The entire slide was screened for areas of greater marking and the proportion of positive cells in each slected area was estimated. The staining intensity and the number of stained cells were recorded separately. The staining intensity was recorded as 1 (negative), 2 (weak), 3 (moderate), and 4 (strong). The p16^INK4a^ expression levels were defined as overexpression and hypoexpression, with a cutoff point of 50% of moderate and/or strong stained cells [53]. The p16^INK4a^ marker was considered positive, if both nuclear and cytoplasmic expressions were present.

#### Survival analysis

Survival at 60 months was assessed in the group of cases, based on the patient's status at the last medical appointment and the recorded death, regardless of the cause. To avoid loss of follow-up, the life status of the participants was checked by the Federal Revenue Service of Brazil, through proof of registration status [54]. Survival was assessed in relation to all variables included in this study, however, only those that presented significant values or clinical importance were included for discussion. In addition, the log-rank test [55] was used to compare survival of CRC patients between the following groups: presence or absence of HPV DNA; p16^INK4a^ expression; tumor origin; tumor size; lymph node involvement; distant metastasis; tumor staging.

#### Data storage and statistical analysis

All data collected were encoded and stored in a data source in Microsoft Excel, version 2013 and IBM SPSS Statistics v.20. The data were analyzed using descriptive statistics in order to generate prevalence estimates with the respective confidence intervals. Associations between qualitative variables were investigated using Fisher's exact test. Possible associations between the expression of p16^INK4a^ and the sociodemographic and clinicopathological aspects of the tumors were assessed by univariate analysis and the calculation of the odds ratio (OR) with a 95% confidence interval (CI). The results were considered statistically significant when p <0.05. For the calculation of survival, the Kaplan-Meier method was used, and to compare the survival curves according to the prognostic factors for colorectal cancer, the log-rank test was used. Death was considered independent of its cause. Since we aimed to evaluate 60 months’ overall survival, the retrospective study that was initiated in 2017, considered patients that were diagnosed until 2010.

## Results

### Descriptive analysis

In total 92 patients with colorectal cancer were analyzed, whose sociodemographic and clinicopathological characteristics are shown in Table 1.

**Table 1:** Sociodemographic, clinical and pathological data of 92 participants with colorectal cancer.

Variables	n	%	Characteristic	n	%
**Age at diagnosis**			**Histological type**		
<50 years	22	23.9	Tubular	78	84.8
≥50 years	70	76.1	Tubulovillous	9	9.8
**Gender**			Mucinous	5	5.4
Feminine	49	53.3	**Treatment**		
Male	43	46.7	Chemotherapy	43	46.7
**Ethnicity**			Radiotherapy	13	14.1
White	51	55.4	Surgery	82	89.1
Brown/Mixed race	37	40.2	**Size of tumor stage (T)**		
Black	4	4.4	Tx	11	12.0
**Marital status**			Tis	1	1.1
Single	44	47.8	T1	8	8.7
Married	48	52.2	T2	32	34.8
Smoker*			T3	37	40.2
Yes	18	23.1	T4	3	3.3
No	60	76.9	**Lymph node metastasis (N)**		
Alcohol consumption*			NX	11	12.0
Yes	9	11.5	N0	45	48.9
No	69	88.5	N1	17	18.5
Proctological status*			N2	19	20.7
With Injury	31	46.9	**Distant metastasis (M)**		
Without Injury	35	53.1	M0	80	87.0
Colonoscopy*			M1a	7	7.6
With Injury	65	90.3	M1b	4	4.3
Without Injury	7	9.7	M1c	1	1.1
**Lesion Location**			**TNM Staging (AJCC)**		
Rectum	48	52.2	Unvalued	11	12.0
Colon	44	47.8	0	1	1.1
**Rectum location**			I	26	28.3
Retosigmoid junction	28	30.4	IIA	14	15.2
Only rectum	5	5.4	IIIA	6	6.5
Rectum upper	8	8.7	IIIB	21	22.8
Rectum medium	4	4.3	IIIC	1	1.1
Rectum down	3	3.3	IVA	7	7.6
**Colon location**			IVB	4	4.3
Sigmoid Colon	20	21.7	IVC	1	1.1
Descending colon	4	4.3	**Registered death**		
Transverse colon	2	2.2	Yes	42	45.7
Ascending colon	16	17.4	Not	50	54.3
Caecum	2	2.3			

* Number of patients with data not informed: Smoker 14; Alcohol consumption 14; Proctological status 26; Colonoscopy 20. Abbreviations: n: Number of patients; AJCC American Joint Committee on Cancer.

Female patients accounted for 53.3% of cases and 76.1% of the cases were over 50 years old. The age of the group varied between 19 and 92 years and the general average was 60 years (± 15.7) at diagnosis.

Most tumors were diagnosed during a colonoscopy (70.7%). The rectosigmoid junction (30.4%) was the main anatomical site affected by the tumors. The most prevalent histological type was the classic tubular adenocarcinoma (84.8%). Dukes' graduation was not reported for all patients and therefore was not analyzed in this study.

Regarding the extent of the disease, most tumors invade the muscle layer itself and reached the pericolic tissues (40.2%). Distant metastases (12 cases) were detected in the liver [4]; bladder [3] and cervix [2]. Other metastases occurred in the small intestine [1], abdominal wall [1] and cecal appendix [1]. Death was recorded for 45.7% of the cases.

### Detection and distribution of HPV genotypes

**Fig 2:**
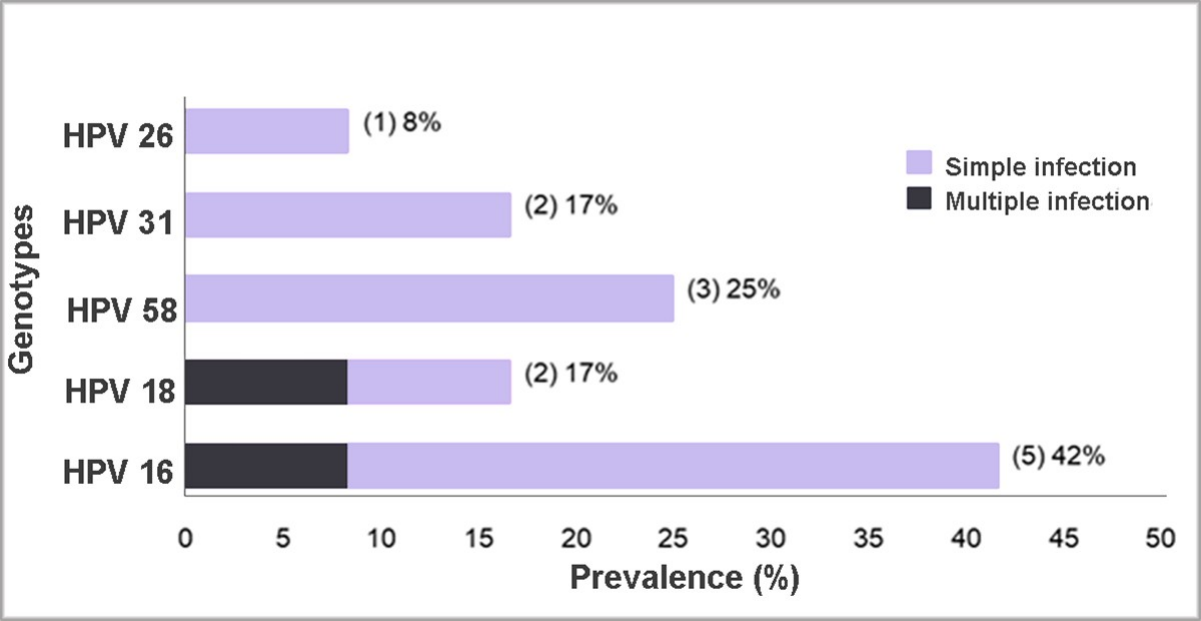
Genotypic prevalence of Human papillomavirus (HPV) in colorectal cancer (CRC).

The prevalence of HPV in CRC was 13% (95% CI 8.4–22.6) (Table 2). The most prevalent genotype was HPV 16 (42%) followed by HPV 58 (25%) and HPV 18 (17%). One sample had multiple HPV 16 and 18 infections (Fig 2). No significant associations were found between the presence of HPV and the variables analyzed. HPV was not detected in the 75 samples of normal tissue adjacent to the tumor or in the 30 samples of precursor lesions (polyps and adenomas) evaluated.

**Table 2:** Univariate analysis of presence of HPV associated with the sociodemographic aspects and the clinicopathological in participants diagnosed with colorectal cancer.

Variable	HPV +		HPV -		*Value p*	**Odds ratio (CI 95%)
	n (12)	%	n (80)	%	*<0.05	
**Age at diagnosis**						
<50 years	3	25.0	19	23.8	1.000	0.9 (0.2–3.8)
≥50 years	9	75.0	61	76.2		
**Sex**						
Feminine	4	33.3	45	56.2	0.214	0.3 (0.1–1.3)
Male	8	66.7	35	43.8		
**Ethnicity**						
White	7	58.3	44	55.0	1.000	0.8 (0.2–2.9)
Brown/Mixed race	5	41.7	36	45.0		
**Marital status**						
Single	7	58.3	37	46.2	0.541	0.6 (0.1–2.1)
Married	5	41.7	43	53.8		
**Smoker**						
Yes	3	33.3	15	21.7	0.423	1.8 (0.4–8.0)
No	6	66.7	54	78.3		
**Alcohol consumption**						
Yes	1	11.1	8	11.6	1.000	1.0 (0.1–9.5)
No	8	88.9	61	88.4		
**Lesion Location**						
Rectum	7	58.3	41	51.2	0.761	0.8 (0.1–3.3)
Colon	5	41.7	39	48.8		
**Histological type**						
Tubular	12	100	66	82.5	0.120	0.8 (0.7–0.9)
Others	0	0.0	14	17.5		
**Size of tumor stage (T)**						
Tis—T2	6	50.0	46	57.5	0.757	1.3 (0.4–4.5)
T3—T4	6	50.0	34	42.5		
**Lymph node metastasis (N)**						
NX—N0	9	75.0	47	58.8	0.354	0.4 (0.1–1.8)
N1—N2	3	25.0	33	41.2		
**Distant metastasis (M)**						
M0	11	91.7	69	86.2	1.000	0.5 (0.6–4.8)
M1	1	8.3	11	13.8		
**TNM Staging (AJCC)**						
0 –II	9	75.0	43	53.8	0.219	2.5 (0.6–10.2)
III–IV	3	25.0	37	46.2		
**Registered death**						
Yes	6	50.00	36	45.00	1.000	0.3 (0.8–1.3)
No	6	50.00	44	55.00		

*Statistically significant values for p<0.05 Fisher exact method.

**Odds ratio (OR) with a 95% confidence interval (CI). Number of patients with data not informed that they were positive for HPV: Smoker 3 (14); Alcohol consumption 3 (14). Abbreviations: n: Number of patients; AJCC American Joint Committee on Cancer.

### Analysis of p16^INK4a^ expression in CRC

All CRC samples were analyzed for p16^INK4a^ expression by immunohistochemistry. Overexpression of p16^INK4a^ was observed in 30.4% of CRC samples. The relationship between the presence of HPV infection and p16^INK4a^ overexpression was investigated (p = 0.07, OR 1.3, CI 95% 0.3–5.4), but no association was observed between these variables, as shown in Table 3. Among the HPV positive CRC cases, three samples presented with p16^INK4a^ overexpression; HPV genotypes in these samples included HPV 58, HPV 31 and HPV 26, and the tumours were located in the rectum with TNM staging (AJCC) I and II.

**Table 3:** Univariate analysis of overexpression (> 50%) of p16^INK4a^ associated with the sociodemographic aspects and the clinicopathological aspects in participants diagnosed with colorectal cancer.

Variable	p16^INK4a^ ≤50%		p16^INK4a^ >50%		*Value p*	**Odds ratio
	n (64)	%	n (28)	%	*<0.*05*	(CI 95%)
**Age at diagnosis**						
<50 years	14	21.9	8	28.6	0.6	0.7 (0.2–1.9)
≥50 years	50	78.1	20	71.4		
**Sex**						
Female	33	51.6	16	57.1	0.6	0.7 (0.3–1.9)
Male	31	48.4	12	42.9		
**Ethnicity**						
White	34	53.1	17	60.7	0.6	0.7 (0.2–1.8)
Brown/Mixed race	30	46.9	11	39.3		
**Marital status**						
Single	32	50.0	12	42.9	0.6	1.3 (0.5–3.2)
Married	32	50.0	16	57.1		
**Smoker**						
Yes	15	27.8	3	12.5	0.1	2.6 (0.6–10.3)
No	39	72.2	21	87.5		
**Alcohol consumption**						
Yes	5	9.3	4	16.7	0.4	0.5 (0.1–2.1)
No	49	90.7	20	83.3		
**Status HPV**						
Present	9	14.06	3	10.71	0.7	1.3 (0.3–5.4)
Absent	55	85.94	25	89.29		
**Lesion Location**						
Rectum	14	21.88	6	21.43	1.0	1.0 (0.3–3.0)
Colon	50	78.12	22	78.57		
**Histological type**						
Tubular	52	81.25	26	92.86	0.2	0.3 (0.6–1.6)
Others	12	18.75	2	7.14		
**Size of tumor stage (T)**						
Tis—T2	40	62.50	12	42.86	0.1	2.2 (0.9–5.4)
T3—T4	24	37.50	16	57.14		
**Lymph node metastasis (N)**						
NX—N0	39	60.94	17	60.71	1.0	1 (0.4–2.5)
N1—N2	25	39.06	11	39.29		
**Distant metastasis (M)**						
M0	56	87.50	24	85.71	1.0	1.1 (0.3–4.2)
M1	8	12.50	4	14.29		
**TNM Staging (AJCC)**						
0 –II	37	57.81	15	53.57	0.8	1.1 (0.4–2.9)
III–IV	27	42.19	13	46.43		
**Registered death**						
Yes	36	56.25	13	46.43	0.4	1.4 (0.6–3.6)
No	28	43.75	15	53.57		

*Statistically significant values for p<0.05 Fisher exact method.

**Odds ratio (OR) with a 95% confidence interval (CI). Number of patients with data not informed that they were p16^INK4a^ >50%: Smoker 4(14); Alcohol consumption 4(14). Abbreviations: n: Number of patients; AJCC American Joint Committee on Cancer.

### Survival analysis

**Fig 3:**
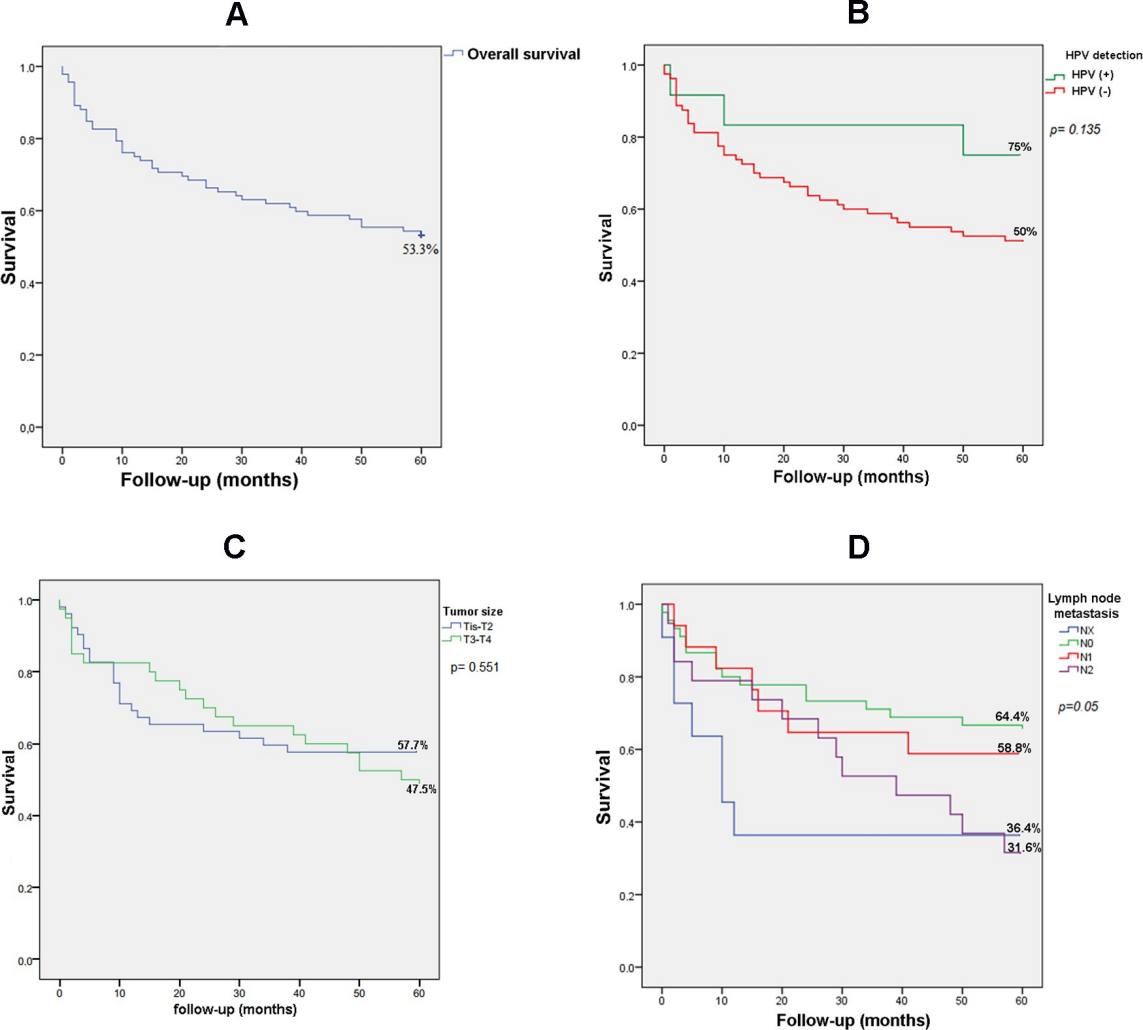
The survival analysis. **A.** Overall 60-month survival for participants with colorectal cancer (Kaplan-Meier method). **B.** 60-month survival for participants with colorectal cancer in relation to the presence or absence of HPV DNA (Kaplan-Meier method). **C.** 60-month survival for participants with colorectal cancer in relation to tumor origin (Kaplan-Meier method). **D.** 60-month survival for participants with colorectal cancer in relation to p16^INK4a^ expression (Kaplan-Meier method).

The overall 60-month survival for patients with colorectal cancer was 53.3% (Fig 3A). The mean follow-up was 40.2 months with a minimum of 0 months and a maximum of 229 months. Survival was not influenced by the presence of HPV (p = 0.135) (Fig 3B). Tumors originating in the rectosigmoid junction had a longer survival than those located in the colon or rectum (p = 0.05) (Fig 3C). The expression of p16^INK4a^ did not influence the survival of the participants with CRC (p = 0.07) (Fig 3D).

**Fig 4:**
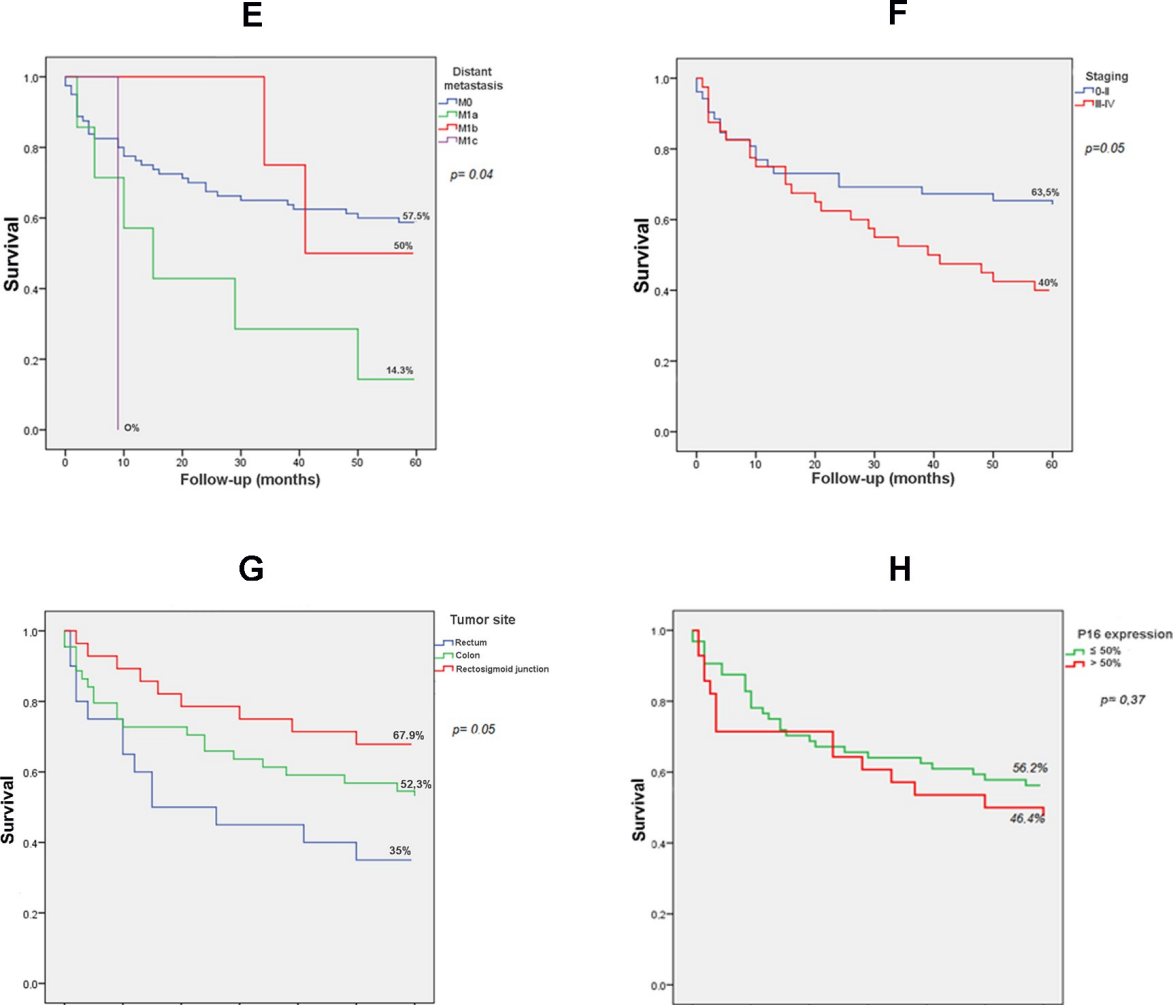
The survival analysis. **E.** 60-month survival for participants with colorectal cancer in relation to tumor size (Kaplan-Meier method). **F.** 60-month survival for participants with colorectal cancer in relation to lymph node involvement (Kaplan-Meier method). **G.** 60-month survival for participants with colorectal cancer in relation to distant metastasis (Kaplan-Meier method). **H.** 60-month survival for participants with colorectal cancer in relation to tumor staging (Kaplan-Meier method).

In the survival analysis due to factors related to tumor stage (Fig 4), survival was influenced by the presence of lymph node metastasis (p = 0.05) and distant metastasis (p = 0.04).

## Discussion

CRC is a highly prevalent tumor and does not present a defined etiology [1], therefore, all risk situations that lead to its development, such as the presence of pathogens, including HPV, need to be investigated [21].

Our results demonstrated the presence of HPV DNA in 13% of the CRC group. However, viral DNA was not detected in the non-cancer tissue adjacent to the tumor or in the precursor lesions of the CRC. In order to establish a causal relationship between HPV and CRC, the virus genome should be detected in tumors and precursor lesions and should be absent in the normal tissue adjacent to the tumor. Therefore, this study does not confirm the hypothesis of an association of HPV with CRC.

The methods used here for the detection of HPV have been previously validated in other tumors associated with the virus, such as penile carcinomas [47] and carcinomas of the anus [48], ensuring the reliability of the results.

There is a high discrepancy in the literature regarding the results of HPV prevalence in CRCs, ranging from 0 to 84.2% [39,41–44,56]. Studies involving cases and controls show a higher frequency of the virus in the tumor tissue, but there are few investigations that use the non-tumor tissue adjacent to the tumor as a control (HPV prevalence ranging from 0% to 13.4%) [43,57–60] as investigated in this study, or even other healthy colonic tissues (HPV prevalence ranging from 0% to 5.8%) [39,57]. With regard to precursor lesions, low prevalence of HPV is described in the reviewed studies (0% to 31%) [40,43,44,56,61–63].

A literature search of the past ten years has resulted in 27 publications that have investigated the association between HPV and CRC, but a consensus on this association has not yet been found [9,40,53,57–60,63–65,66–75,76–82]. In the studies evaluated, the prevalence of HPV in CRCs ranged from 0 to 57.1%. Among these studies, only four investigated the non-cancer tissue adjacent to the tumor [57–60] and only one detected HPV in these sites (8.7%) [59]. On the other hand, eight studies [57,67,68,71,73,76,79,81] investigated HPV in CRC precursor lesions, including polyps and adenomas, and three of them were positive for HPV detection, with a prevalence ranging from 5.7% to 27.7% [67,68,71].

Different methods were used by the 27 studies mentioned above [9,40,66–75,53,76–82,57–60,63–65]. Most of them analyzed samples preserved in paraffin and performed HPV detection by PCR with GP5/6 and MY09/11 primers [59,60,81–83,69–73,75,78,79]. These primers amplify larger HPV DNA fragments (150bp to 450pb), and can be a problem when paraffin embedded samples are used, since DNA obtained from such samples are more prone to DNA degradation compared to that obtained from frozen or fresh samples [46]. In addition, these primers present variable sensitivity for detecting different types of HPV and low specificity for oncogenic HPVs genotypes [84,85].

In the present study, we used SPF10 primers that amplify small fragment of DNA (65bp) from a large number of HPV genotypes, enabling HPV genome detection even in those samples with DNA integrity partially compromised. The INNOLIPA method used in our study has been widely used for HPV detection and genotyping by several groups investigating different tumors potentially associated with HPV [86–88], and our experience with this method has been already demonstrated in previous studies [47,48].

Standardization of HPV detection and genotyping methods is essential to ensure the reliability of the results obtained in different studies. Greater attention must be given to the pre-analytical stages, including formaldehyde fixation techniques and sample handling, in order to prevent DNA degradation and also to avoid contamination between samples. In this study, all the tumor specimens evaluated were approximately the same age and all samples were fixed in formaldehyde and embedded in paraffin by the same laboratory, following a standardized protocol. By replacing the razors and cleaning the microtome with 70% ethanol at each new block, the possibility of contamination between samples was reduced, and in order to guarantee the quality of the isolated DNA samples, both spectrophotometry analysis and two endogenous control amplification steps were performed, including a housekeeping gene chosen by the laboratory (GAPDH gene) and the endogenous control of the INNO-LiPA kit (endogenous human HLA-DBP1 gene).

The largest case control publication carried out in the last ten years on the detection of HPV in CRC was conducted in France by Vuitton (2017) and similar to this study, it included two control groups, comprising 217 adenocarcinomas; 210 neoplasia-free tissues adjacent to the tumor of the same patient, and 40 samples of the sigmoid colon of patients with colonic diverticulosis. Unlike the results presented here, there was no detection of HPV DNA in any of the 467 cases and the methodology was based on qRT-PCR with primers for the E6 gene of HPV 16 and 18 [57].

Three meta-analyzes on the detection of HPV in CRC have already been published, with results of combined prevalence of 11.2% (95% CI, 4.9–19.6) [43]; 31.9% (95% CI, 19.3–47.9) [41], and 45% (95% CI, 0.36–0.53) [56]. Baandrup (2014) included eight case-control studies and the overall prevalence of HPV was 36.8% in adenocarcinomas, 5.1% in adenomas; 13.4% in non-cancer tissues adjacent to the tumor and 1.6% in tissues free of disease. Damin (2013) described a 10.04% increase in the risk (95% CI 3.67–27.46) for CRC in relation to the presence of HPV. In the study by Zhang (2018) only publications with the Chinese population were included and the prevalence of the virus in tumor tissues and adenomas was higher than that observed in controls OR 10.78% (95% CI 4.22–27.53), highlighting the association of HPV with CRC in the Chinese population.

The results of the meta-analyzes should be interpreted with great care, mainly due to the heterogeneity of the samples, HPV detection methods, geographical differences and possible contamination of the tumor samples during clinical processing, observations that the authors themselves highlighted in their studies [41,43,56].

Hypothetically, the wide difference in the prevalence of HPV infection observed in CRC samples could be partially explained by the geographical variation, as reported in two metanalyses [41,43] demonstrating higher values of HPV infection in colorectal cancers from South America, Asia and the Middle East, ranging from 32% to 45%, and lower values of HPV infection in CRC (3% or less) from North America, Europe and Australia [43]. However, more recent publications contradict those findings demonstrating higher prevalences of HPV infection in CRC from the European continent (12.3% to 16.9%) [40,59] and North America (23.8% to 42.2%) [64,68,69].

It is important to highlight that, even within the same geographic region, a substantial heterogeneity in the prevalence of HPV infection in CRCs might be observed, as shown by a meta-analysis including only publications from China [56]. In other words, the differences observed in the HPV infection prevalence in colorectal cancers can not necessarily be explained by geographical characteristics, but it seems to reflect the individual particularities of each study, such as methodological issues, which include detection techniques, sample preparation, measures to avoid contamination, type of material used for analysis, age of the populations analyzed and anatomical location of the tumor [39,89].

In Brazil, geographic differences do not justify the discrepancy observed in the prevalence of HPV infection in CRCs. So far, only two studies investigated the presence of HPV DNA in CRCs, with a prevalence of 28.4% in the first study carried out in the North of the country, a region considered very poor and with the worst overall health indicators in the country [71], and the second one, carried out in the Southern of the country, a region considered economically developed, demonstrating a prevalence of 63.9% of HPV DNA in colorectal adenocarcinomas [61]. It’s important to mention that the two studies were methodologically different.

Regarding the distribution of the genotypes detected in our study, HPV 16 was the most frequently detected, and multiple HPV 16 and 18 infections was observed in only one case. HPV 16 is the most commonly detected genotype, both in tumor samples [71,75,77,78,80] as well as non-tumor samples [67–69].

In this study, the presence of HPV was not different in relation to the location of the tumor in the colon or rectum, as previously reported by other authors [61,90,91]. Interestingly, some authors cite that one of the possible routes for HPV to reach the colon or rectum would be the ascending or retrograde route, through sexual intercourse or inoculation with fomites or even through the preventive colonoscopy [53,83,92].

The potential prognostic role of HPV in colorectal cancers is not known, and the number of publications that correlated the virus with the stage and classification of the tumors is still small [74,80]. In the present analysis HPV detection did not show any significant association with clinicopathological characteristics and survival of the patients with CRC.

To investigate the possible role of HPV in colorectal carcinogenesis, it is necessary not only to demonstrate the presence of viral DNA in tumors, but also the transcriptional activity of the virus and tissue proteins in response to infection. For this purpose, the expression of the tumor suppressor gene p16^INK4a^ was investigated and its overexpression was observed in 30.5% (n = 28) of CRC cases, with only three of these cases being positive for HPV detection, suggesting that in fact the virus was not involved in the carcinogenic process of these tumors, and that its presence is considered incidental.

The relationship between p16^INK4a^ expression in the CRC and the presence of HPV is still poorly investigated [53]. The overexpression of p16^INK4a^ detected by immunohistochemistry is considered a sensitive marker for the presence of transcriptionally active infections by high-risk HPV genotypes, mainly in cervical and oropharyngeal tumors related to the virus [36,93–95]. Generally, in these tumors, increased levels of viral oncoproteins E6 and E7 are observed, acting on important molecular mechanisms of carcinogenesis and resulting in increased expression of p16^INK4a^ [26,38,96].

A key event for the increase in p16^INK4a^ due to HPV infection is the inactivation or degradation of retinoblastoma protein (pRb) by the high-risk HPV viral oncoprotein E7. The pRb signaling pathway is regulated by the tumor suppressor protein p16^INK4a^. In addition, p16^INK4a^ also indirectly regulates the p53 pathway [97]. Inactivation or decrease in pRb activity initiates a feedback loop that results in the activation of senescence-promoting pathways that include increased expression of p16^INK4a^ [37,98–100]. Therefore, E7-mediated inactivation of pRb results in elevated levels of p16^INK4a^ in HPV-infected cells.

Recently a Polish study evaluated the presence of HPV DNA in a group of 120 individuals with adenocarcinomas of the rectum, demonstrating only three HPV DNA positive tumors (2.5%), and all the three cases were negative for p16^INK4a^ overexpression [89]. In our study all tumor samples, positive or negative for HPV detection, were tested for p16^INK4a^ expression, and it was verified that some of the HPV DNA negative tumors also presented with p16^INK4a^ overexpression. Different mechanisms independent of HPV infection can explain p16^INK4a^ overexpression, such as genetic changes in the Rb gene, mutations in the p16^INK4a^ gene itself and induction of senescence by different oncogenic mechanisms [101].

An additional investigation on the expression of p16^INK4a^ and the clinicopathological aspects of CRC was also conducted in our study, however the results did not show any significant association between p16^INK4a^ and the variables analysed and not even survival was influenced by the expression of p16^INK4a^.

A recent meta-analysis investigated the prognostic relevance of p16^INK4a^ in CRC and suggested that its overexpression could be correlated with the Dukes stage, the presence of lymph node metastasis, and the TNM stage (only in Caucasians), implying in a relevant role for p16^INK4a^ protein in the development of CRC [102]. However, the study emphasized several limitations of the publications including inconsistent results in Asian studies and the sample sizes in others.

Prognostic aspects of patients with CRC were also assessed in our study, with survival being influenced by higher staging (p = 0.05), lymph node involvement (p = 0.05) and distant metastasis (p = 0.04). These parameters are still the most significant to be considered for the treatment of CRC. The patients in this study had an overall survival of 53.3%, indicating the still late diagnosis of these tumors and the lack of more effective screening programs in the country. In Brazil, although the public health system offers comprehensive primary health care services, the country does not have a national government policy or program addressing CRC prevention and control [103]. Unfortunately, there is still much to be discussed about CRC screening and prevention strategies in the country.

Some limitations were found during the research and ended up influencing the sample size, among them, the lack of complete and relevant information in the medical records, such as sexual behavior and the availability of sufficient tumor tissue blocks for analysis. Even so, our data adequately represented the reality of participants with colorectal cancer in the Midwest region of Brazil, due to the wide regional service area of the referral hospital.

The low frequency of HPV in CRC, its absence in control groups, and the lack of relationship between the expression of p16^INK4a^ and the detection of the viral genome in this study rule out a possible carcinogenic role of HPV in CRC. It is important to note that recent studies with large populations show little or no detection of HPV in CRC [57,60,77,79,104,105], and even studies that assessed viral load and the viral integration event in CRC do not demonstrate the causal role of the virus in these tumors [40,58,63,69,74].

## Conclusions

In this study, HPV was not associated with colorectal cancer, nor did it influence its clinical or prognostic aspects. In addition, the expression of p16^INK4a^ was not relevant as a marker of transcriptionally active HPV infection in CRC. Based on the prognostic aspects evaluated, the patients' survival was influenced by the higher degree of staging, lymph node involvement and distant metastasis, suggesting that these parameters are still the most significant and valid for defining the treatment of CRC.

## Supporting information

S1 File Data-base-supplementary. (XLS)

S2 File DNA-quantity-and-quality-supplementary. (XLS)
